# Androgen-related expression of G-proteins in ovarian cancer

**DOI:** 10.1038/sj.bjc.6605153

**Published:** 2009-07-21

**Authors:** L A Sheach, E M Adeney, A Kucukmetin, S J Wilkinson, A D Fisher, A Elattar, C N Robson, R J Edmondson

**Affiliations:** 1 Northern Institute for Cancer Research, Newcastle University, Newcastle, UK; 2 Northern Gynaecological Oncology Centre, Queen Elizabeth Hospital, Gateshead, UK

**Keywords:** ovarian cancer, androgens, G-proteins, gene expression

## Abstract

**Background:**

Epidemiological and *in vitro* data implicate androgens in the aetiology of ovarian cancer, but the mechanisms by which this is mediated are unclear. In this study, we wished to examine the effects of androgens on gene expression in ovarian cancer.

**Methods:**

The expression of androgen receptor (AR) in OVCAR3 and OSEC2 cells was confirmed using immunoblotting and response to androgens was measured using flow cytometric assessment of S-phase fraction. The differential gene expression between androgen stimulated and unstimulated OVCAR3 ovarian cancer cells was examined with a cDNA microarray. The upregulation of a subset of these genes was then confirmed with reverse transcriptase PCR in both OVCAR3 and OSEC2, an ovarian epithelial cell line. Finally, the clinical significance of this upregulation was investigated by examining the expression of Rab25 and Rab35, two G-protein-related molecules in an ovarian cancer tissue microarray (TMA).

**Results:**

OVCAR3 and OSEC2 cells were shown to express the AR and showed an increase in S-phase fraction in response to androgen treatment. Treatment of OVCAR3 cells with androgen resulted in a significant upregulation of 121 genes. These findings were confirmed for a subset of seven monomeric G-protein-related genes in both OVCAR3 and OSEC2 cells. After staining for Rab25 and Rab35, the majority of TMA sections examined showed expression for Rab25 (92%) and Rab35 (95%). The expression of Rab25 correlated with histological grade, and expression was higher in endometrioid (median histoscore 10.5) than serous (7.5) or mucinous (5.3) tumours. The expression of Rab25 correlated positively with AR expression supporting its role as an androgen responsive gene in ovarian cancer.

**Conclusions:**

These results suggest that androgens can effect expression of the oncogenic GTPases in ovarian cancer. We propose that the androgen responsive Rab35 may have clinical importance as a biomarker of AR function.

There is a wealth of evidence to suggest that many epithelial ovarian cancers (EOC) are hormonally related (Lukanova and Kaaks, 2005). Epithelial ovarian cancer is thought to arise from the ovarian surface epithelium (OSE), a single layer of cells overlying the ovary, which is in close proximity to the hormone-producing cells of the ovary. Many cancers are proposed to arise from invaginations of the OSE, which follow ovulation and create inclusion cysts (Auersperg *et al*, 2001). The cells lining these cysts lack a tunica albuginea, and are therefore exposed to even higher levels of steroid hormones, including oestrogens, progesterones and androgens.

Epidemiological evidence suggesting that hyperandrogenic states are associated with the development of EOC (Schildkraut *et al*, 1996; Wang and Chang, 2004; Gaducci *et al*, 2005) has led to the development of the androgenic theory of ovarian cancer development (Risch, 1998). Androgens exert their effects through activation of the androgen receptor (AR), a type I nuclear hormone receptor, involved in gene transcription. The action of AR is controlled by a series of coregulators (Gnanapragasam *et al*, 2000).

We have previously shown that OSE cells express the AR and respond to androgen stimulation resulting in increased proliferation and protection from apoptosis (Edmondson *et al*, 2002), others have shown that the majority of ovarian cancers continue to express the AR (Kuhnel *et al*, 1987; Ilekis *et al*, 1997; Lee *et al*, 2005). Other phenotypic effects of androgens on ovarian cancer cells and how these are mediated are poorly understood, although it has been shown that androgen treatment allows these cells to escape the growth inhibitory effect of transforming growth factor *β* (TGF*β*) by downregulating the expression of the TGF receptors, TGF*β*R I and II (Evangelou *et al*, 2000). Androgen stimulation also results in changes in expression of the steroid hormone coregulators, SRC-1, ARA70 and AIB1, although this cofactor is lost in the neoplastic transformation of OSE cells to EOC (Evangelou *et al*, 2003).

Here, we investigate the effects of androgens on gene expression in cells expressing the AR, and investigate the clinical relevance of the subsequently identified androgen responsive genes.

## Materials and methods

### Tissue microarray

The Newcastle ovarian cancer tissue microarray (TMA) has been described previously (Wilkinson *et al*, 2008). Briefly, the TMA comprises 154 cases of histologically confirmed EOC, with accompanying clinical data available for each case. All tissue samples were taken from sequential patients who underwent primary surgery between 1995 and 2000 at the Northern Gynaecological Oncology Centre, Gateshead, UK, with appropriate ethical approval. All patients underwent maximal-effort primary cytoreductive surgery followed by platinum-based chemotherapy with or without paclitaxel.

### Cell lines and cell culture

OVCAR3 cells were obtained from the American Tissue and Cell Collection and maintained in RPMI-1640 medium (Sigma-Aldrich, Poole, UK) with the addition of 20% (v/v) foetal bovine serum (Sigma-Aldrich) and 1% L-Glutamine (20 mM) (Sigma-Aldrich).

OSEC2 cells have been previously described (Davies *et al*, 2003). Briefly this cell line was derived from a primary culture of normal ovarian epithelial cells created following an oophorectomy for a non-malignant pathology. Cells were immortalised using hTERT and the temperature-sensitive form of SV40 large T antigen. OSEC2 cells are maintained at the permissive temperature of 30°C in RPMI-1640 medium (Sigma-Aldrich) with the addition of 10% foetal bovine serum (Sigma-Aldrich) and 1% L-Glutamine (20 mM) (Sigma-Aldrich).

### Cell proliferation assays

Cell cycle analysis was carried out using flow cytometry. After stimulation with dihydrotestosterone (DHT), cells were harvested, washed in PBS and resuspended in a propidium iodide (PI) solution (RNAase (1%w/v), PI (18%w/v), FCS (2%v/v) in 5% Triton) for 30 min before being analysed on a Becton-Dickinson FACScan Flow Cytometer (BD Becton-Dickinson UK Limited, Oxford, UK). Data were analysed using WinMidi software (Scripps Institute, San Diego, CA, USA).

Cell proliferation was measured using a sulphorhodamine B (SRB) assay. Briefly, cells were seeded in 96-well plates, washed with PBS and quiesced in serum-free medium for 48 h before DHT treatment. Cells were fixed with 50% trichloroacetic acid and stained with 0.4% SRB solution before the addition of10 mM Tris. All plates were analysed on a spectrophotometer at 570 nm. Data presented are the results of six independent experiments.

### Western blotting

Cells were harvested in lysis buffer (1%Triton-X, 0.1% sodium dodecyl sulphate, 0.5% sodium deoxycholate) and were fractionated using SDS-PAGE in a mini-protean III system (BioRad, Herts, UK) followed by electrotransfer onto nitrocellulose membrane (Hydrobond C, Amersham Biosciences, Amersham, UK).

Membranes were incubated overnight at 4°C with mouse monoclonal anti-human AR antibody, at a concentration of 1 : 1000 (clone AR441, Dako, Glostrup, Denmark). Blots were reprobed with anti-*α*-tubulin antibody (1 : 2000)(Dako) to confirm equal loading of protein.

The immunocomplex was detected using appropriate secondary antibodies and visualised using enhanced chemiluminescence detection system (ECL)(Amersham Biosciences).

### cDNA microarray analysis and real-time RT-PCR

Total RNA was extracted from DHT stimulated and unstimulated OVCAR3 cells using a commercially available kit (RNeasy, Qiagen, Crawley, UK). Concentrations of RNA >1000 ng *μ*l^−1^ were used for cDNA microarray analysis (Human Genome U133 Plus 2.00 gene chip, Affymetrix, High Wycombe, UK). Data were normalised and analysed using Genespring software (Agilent Technologies, Edinburgh, UK). A twofold change in gene expression compared with unstimulated cells was considered significant. Ontological analysis was performed using the Netaffx database (http://Affymetrix.com/). Quantitative real-time RT-PCR analysis was performed using Jumpstart SYBR Green master mix (Sigma-Aldrich) and specific primers (VH Bio Ltd, Gateshead, UK) were designed using Primer Express software (version 2.0, Applied Biosystems Inc. Foster City, CA, USA). Expression levels were normalised against a housekeeping gene, GAPDH. All QPCR experiments were performed on a 7900-HT sequence detection system and integral SDS software (Applied Biosystems, Warrington, UK) was used for analysis.

### Immunohistochemistry

Tissue microarray sections were deparaffinised and rehydrated through a series of graded alcohols. Antigen retrieval was achieved by the pressure cooker method in 0.01 M citrate buffer (pH 6.0). Slides were then washed in PBS before being treated with a 1.7% hydrogen peroxide/methanol solution. A total of 10% blocking serum was applied for 20 min at room temperature before application of the primary antibody overnight at 4°C. Monoclonal mouse anti-human AR antibody was used at a concentration of 1 : 20 (clone 441, Santa Cruz, Heidelberg, Germany). Monoclonal mouse anti-human Rab25 antibody was used at a concentration of 1 : 500 (clone 3F12F3, ProMab Biotechnologies, Richmond, CA, USA). Polyclonal rabbit anti-human Rab35 antibody was used at a concentration of 1 : 20 (Proteintech, Manchester, Peterborough, UK). Primary antibody was detected with an appropriate biotin-conjugated secondary antibody (Vector Laboratories, Peterborough, UK). Slides were then developed using an avidin–biotin–peroxidase complex kit (Vectastain, Vector Laboratories), incubated with 3′,3′-diaminobenzidine (Sigma-Aldrich), counterstained using Harris' haematoxylin before being dehydrated and mounted. Negative control sections were incubated with secondary antibody alone. Tissue controls included prostate, breast and thyroid tissues for AR, Rab25 and Rab35, respectively. Each core was independently scored by two of the authors (EMA and RJE) using a modified *H*-score (Leake *et al*, 2000). The *H*-score is a multiplicative score generated by two parameters, namely maximal intensity of staining and percentage area of the section stained. Scores range from 0 (no staining throughout the section) to 18 (high intensity staining throughout the whole section). Any differences of opinion were mutually resolved, For the purpose of analysis, scores were further categorised as low (less than median score) or high (greater than median score).

### Statistics

Statistical analysis was performed using SPSS version 11.0 (SPSS Inc., Chicago, IL, USA). Statistical significance was evaluated using the log-rank test, non-parametric analysis by Kruskal–Wallis, Kaplan–Meier analysis for survival and Cox regression for multivariate analysis. A *P*-value of <0.05 was considered statistically significant. Correlation was analysed using Spearman's correlation.

## Results

### OVCAR 3 and OSEC2 cells express AR and are responsive to androgen stimulation

**Figure 1:**
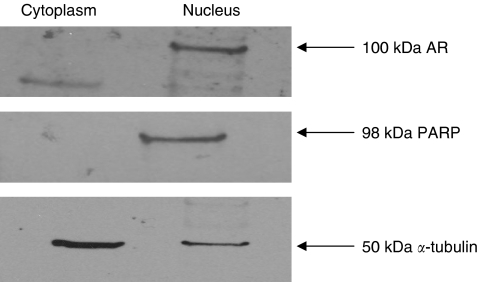
Western blot showing nuclear and cytoplasmic fractions of OVCAR3 cells after treatment with 10 nM DHT. Androgen receptor (AR) is expressed in the nuclear fraction, as confirmed by the presence of PARP, in contrast to the cytoplasmic fraction suggesting the presence of functionally active protein.

The presence of AR in OVCAR3 and OSEC2 cells was confirmed using western blot of nuclear and cytoplasmic extracts after DHT treatment. Androgen receptor protein was found to be exclusively nuclear, in keeping with a functional protein (Figure 1).

**Figure 2:**
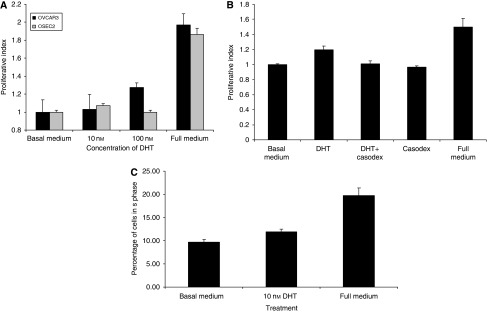
Treatment with DHT results in an increase in cell proliferation in both normal and malignant ovarian cells. (**A**) OSEC2 and OVCAR 3 cells were stimulated for 24 h with DHT before analysis using an SRB assay. Both cell lines show increases in cell proliferation after DHT stimulation of up to 24 h. Increased cell proliferation is shown using a dose of 10 nM in the OSEC2 cells, but a dose of 100 nM in OVCAR3 cells. (**B**) OVCAR3 cells were treated for 24 h with 10 nM DHT in the presence and absence of the specific AR inhibitor, Casodex, before analysis using an SRB assay. The androgen-induced stimulation is abrogated by the addition of Casodex. Casodex alone had no effect on cell proliferation. (**C**) OVCAR3 cells were treated with DHT for 24 h and the S-phase fraction analysed using propidium iodide incorporation. Cells stimulated with DHT show a dose-dependent increase in the S-phase fraction compared with non-treated cells.

Stimulation of OSEC2 and OVCAR3 cells with increasing doses of DHT for 24 h resulted in an increase in cell proliferation of up to 25% over unstimulated cells (Figure 2A). This observed increase was abrogated with the addition of the AR-specific antagonist, Casodex, suggesting that this effect is AR dependent (Figure 2B). Stimulation of OVCAR3 cells with increasing doses of DHT for 24 h results in an increase in the S-phase fraction over unstimulated cells (Figure 2C).

### Androgen stimulation promotes differential expression of multiple genes

Messenger RNA was extracted from OVCAR3 cells that were either untreated or exposed to 10 nM of DHT for 2 or 8 h. Microarray analysis was then performed using the Affymetrix u133 gene chips (Affymetrix) and the expression was compared with that of non-stimulated cells. Results were normalised and analysed using Genespring software. Stimulation with DHT resulted in greater than twofold upregulation of 33 and 105 genes at 2 and 8 h, respectively, and downregulation of 1 and 16 genes at 2 and 8 h, respectively. Seven genes were upregulated at both 2- and 8-h time points.

Ontological analysis of the upregulated genes indicated that the majority were related to gene transcription, but some genes associated with proliferation and G-protein signalling were also found to be increased compared with the untreated controls (Table 1).

Upregulation of mRNA expression was confirmed in a subset of eight G-protein-related genes using RT-PCR. Changes in mRNA levels of G-protein-related genes in both OVCAR3 and OSEC2 cells after stimulation with 10 nM of DHT for 2, 4, 8, and 12 h confirmed the data obtained from the microarray experiment for the subset of genes tested (Table 2).

### Rab25, Rab35 and AR are expressed by ovarian cancers

In light of the finding that G-proteins were upregulated in response to androgen stimulation, we decided to further investigate the expression of two of these proteins in clinical ovarian cancer samples. Rab35 was chosen as it was the most highly upregulated gene in the cDNA microarray experiment, and Rab25 was chosen as this protein has been linked with ovarian cancer (Cheng *et al*, 2004a, 2004b; Cheng *et al*, 2005).

**Figure 3:**
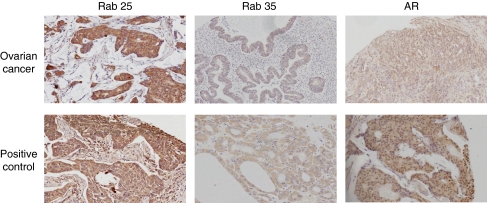
Rab25, Rab35 and AR expression in clinical samples. Rab25, Rab35 and AR protein expression levels were determined using immunohistochemical techniques for 154 epithelial cancer samples arrayed on a TMA. Representative sections are shown demonstrating predominantly cytoplasmic staining for Rab25 and Rab35, and nuclear staining for AR. Breast, thyroid and prostate sections were used as positive controls for Rab25, Rab35 and AR, respectively.

Clinical characteristics of the patient samples included in the TMA have been previously published (Wilkinson *et al*, 2008). The TMA was stained for AR expression (Figure 3). As expected, staining was predominantly nuclear, 86% of samples showed positivity for AR. Androgen receptor scores showed no correlation with FIGO stage, histological subtype, residual disease, preoperative CA125 levels or overall survival.

**Figure 4:**
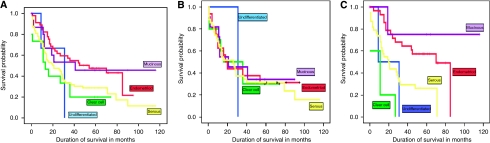
High expression of Rab25 predicts survival by histological type. (**A**) Kaplan–Meier survival curves of all patients included in this study show differential expression by histological type. (**B**) Kaplan–Meier curves of only those tumours showing low expression of Rab25 shows no difference in survival (**C**) Kaplan–Meier curves of only those tumours showing high expression of Rab25 attenuates this finding with improved survival in mucinous and endometrioid tumours compared with serous and clear cell cancers (*P*=0.043).

The TMA was then stained for Rab25 expression (Figure 3). The majority of staining was cytoplasmic, with 92% of samples showing positivity for Rab25. Rab25 scores were higher in endometrioid tumours (median score 10.5) compared with serous (median score 7.5), mucinous (median score 5.3) and clear cell (median score 5.8) tumours, but there was no correlation with FIGO stage or residual disease (Table 3). Overall, Rab25 expression did not correlate with survival, but when classified by histological subtype, high expressors (expression greater than the median score (7.5)) showed variation by histological subtype unlike low expressors, which showed no variation in survival (Figure 4).

The TMA was stained for Rab35 expression (Figure 3). As expected, staining was entirely cytoplasmic, 95% of samples showed positivity for Rab35. Rab35 scores showed no correlation with FIGO stage, histological subtype, residual disease, preoperative CA125 levels or overall survival when analysed by high and low expressors (high expressors defined as expression greater than the median score (4.7)).

Finally, correlation was investigated between scores for AR, Rab25 and Rab35 expression. No significant correlation was found between Rab25 and AR expression, but a significant positive correlation was seen between Rab35 and AR expression, (*r*=0.23, *P*<0.01).

## Discussion

We have described here the effects of the androgen DHT on both cell proliferation and gene expression in ovarian cancer cells. This physiologically important hormone has been linked with ovarian cancer; however, this is the first study investigating the effects of acute exposure to androgens on gene expression. The findings are therefore likely to differ from other studies in which gene expression was analysed after chronic exposure to androgens (Motamed-Khorasani *et al*, 2007).

We have shown that, in addition to some predicted increases in gene expression in genes encoding proteins related to transcription regulation and cell cycle control, there was also an increase in the expression of several proteins related to G-protein signalling. The G-proteins are a superfamily of ubiquitous signalling molecules. They are involved in intracellular signalling from G-protein-coupled receptors (GPCR), other receptors and also independently (Andreeva *et al*, 2007). There are two distinct groups of G-proteins: the large (heterotrimeric) form, and the small (monomeric) form, otherwise known as the Ras superfamily of GTPases. The large G-proteins exist at the cell surface next to the GPCR and are made up of three sub units: *α*, *β* and *γ*. Ligand binding and exchange of GDP for GTP on the *α*-subunit allow signal transduction, which is terminated by GTP hydrolysis back to GDP. The small GTPases exist unbound to the GPCR. They contain an *α*-subunit with a structurally homologous GTPase domain to that of the large heterotrimeric G-proteins (Oldham and Hamm, 2006). Similarly, their action depends upon the binding of GTP. Once activated, they initiate signalling cascades, phosphorylating downstream proteins and causing an effect. They are split further into five families: Ras, Rho, Rab, Ran and Arf. We have shown that when stimulated with androgens, a number of G-proteins are upregulated in OVCAR3 cells. We investigated two of the small G-proteins further: Rab25 and Rab35. We chose to examine Rab35 as this was the most differentially expressed gene upon androgen stimulation, and Rab25 as a comparison as this was only marginally altered in response to androgens.

Rab25 is a small GTPase of 28 kDa, localised in epithelial cells. It is involved in protein trafficking from the cell membrane to cytoplasmic vesicles (Cheng *et al*, 2005), and is known to be involved in proliferation, protection from apoptosis and invasion in ovarian cancers (Yang *et al*, 2006). It has been shown to be upregulated in 80% of ovarian cancers and its expression correlates with a worse disease-free survival in both ovarian and breast cancers (Cheng *et al*, 2004a, 2004b).

Of interest is the observation that Rab25 expression is only marginally upregulated after exposure to androgen, as an androgen response element in the antisense strand of the Rab25 gene has been found (Louro *et al*, 2007). It is possible that effects observed on Rab25 expression are mediated by an indirect mechanism, perhaps through TGF*β* receptors, which have been shown to interact with the AR in ovarian cancer (Evangelou *et al*, 2000). Furthermore, it has been proposed that endometrioid tumours develop as a result of TGF*β*R-II (Lynch *et al*, 1998) and MSH2 (Fujita *et al*, 1995) mutations rather than mutations of k-Ras (Enomoto *et al*, 1991). Our finding, therefore, that Rab25 expression is greatest in endometrioid tumours does not rule out a possible indirect interaction between androgen stimulation, the TGF*β* receptors and Rab25.

Another small GTPase, Rab35, was the most upregulated upon androgen stimulation in our experiments. Little is known about this protein, although it is thought to be involved in endocytic recycling (Kouranti *et al*, 2006) and may play a role in the modulation of p53 by regulating the p53-related protein kinase, PRPK, giving it oncogenic potential (Abe *et al*, 2006). We have not shown any clinical significance related to Rab35 expression, although 95% of the tumours examined showed expression of the protein. The finding that Rab35 and AR expression correlate supports the concept that *Rab35* is an androgen responsive gene, and given the timescale of upregulation after androgen stimulation, it is likely that this is a direct effect. Further work is required to investigate the functional role of Rab35 in ovarian cancer, but this gene may have use as a biomarker of AR function in ovarian cancer, thus being able to predict those patients who are likely to respond to antiandrogen therapy.

Taken together, these data suggest that androgen treatment has downstream effects on small G-protein signalling cascades, however, this effect only occurs in a subset of ovarian cancers. Further work is required to investigate these effects, and to identify whether there is a subset of ovarian cancers in which this is important, which may be suitable for a targeted antiandrogen therapy.

## Figures and Tables

**Table 1:** Ontological classification of genes upregulated in OVCAR3 cells after exposure to 10 nM DHT

**Ontological group**	**2 h**	**8 h**	**Both**	**Total**
Transcription	19	47	3	63
Proliferation	7	14	1	20
G-protein signalling	3	12	2	13
Apoptosis	1	17	0	18
Ubiquitination	1	7	1	7
Cell adhesion	2	8	0	10
Total	33	105		131

DHT=dihydrotestosterone.

**Table 2:** Changes in expression of G-proteins after 8-h stimulation by 10 nM DHT

			**Maximal fold change upregulation**		
			**OVCAR3**		**OSEC2**
**Gene**	**Accession number**	**Brief description of known function**	**Affymetrix**	**RT-PCR**	**RT-PCR**
*GNA13*	NM_006572	*α*-Subunit of heterotrimeric G-protein 13	2.2	5.5	146.1
*ELKS*	AB029004	RAB6-interacting protein 2. Regulator of NF-*κ*B signalling	2.9	2	1.4
*GSTP1*	BE544748	G to S-phase transition protein 1	2.5	2	7.8
*RERG*	AW294092	Monomeric G-protein	2.9	3	5.2
*Rab25*	NM_020387	Monomeric G-protein. Involved in vesicle trafficking	1.05	1.42	ND
*Rab45*	NM_152573	Monomeric G-protein. Involved in vesicle trafficking	2.5	2.5	8.6
*Rab35*	NM_006861	Monomeric G-protein. Involved in vesicle trafficking	5.4	13	13.1

DHT=dihydrotestosterone; NF-*κ*B=nuclear factor *κ*B; RT-PCR=reverse transcriptase PCR.

**Table 3:** Correlation of expression of Rab25 and Rab35 with clinical parameters

		**Rab25**		**Rab35**		**AR**	
**Variable**	***n* (%)**	**Median**	***P***	**Median**	***P***	**Median**	***P***
Available to score	154						
							
*Stage*			0.78		0.76		0.6
I	27 (17.5)	7		4.7		7.25	
II	14 (9)	11.3		4.5		11.25	
III	91 (59)	7.5		4.7		7.5	
IV	18 (12)	7.8		5.7		8.25	
Missing	4 (3)						
							
*Grade*			0.49		0.58		0.75
1	38 (25)	7		5.5		7.5	
2	41 (27)	7.8		4		8.5	
3	75 (49)	7.5		4.7		7.5	
Missing	0						
							
*Histological subtypes*			0.001		0.32		0.02
Serous	73 (47)	7.5		5		7.5	
Mucinous	16 (10)	5.3		3.7		5.25	
Endometrioid	47 (31)	10.3		5.3		10.5	
Clear cell	15 (10)	5.8		6.1		6	
Missing	3 (2)						
							
*Residual disease*			0.56		0.32		0.4
None	54 (35)	8.5		4.7		9	
⩽1 cm	30 (19)	8.5		4.8		8.5	
>1 cm	64 (42)	7		5		7	
Missing	6 (4)						

AR=androgen receptor.

## References

[bib1] Abe Y, Takeuchi T, Imai Y, Murase R, Kamei Y, Fujibuchi T, Matsumoto S, Ueda N, Ogasawara M, Shigemoto K, Kito K (2006) A small Ras-like protein Ray/Rab1c modulates the p53-regulating activity of PRPK. Biochem Biophys Res Commun 344(1): 377–385 16600182 10.1016/j.bbrc.2006.03.071

[bib2] Andreeva A, Kutuzov M, Voyno-Yasenetskaya T (2007) Scaffolding proteins in G-protein signaling. J Mol Signal 2(1): 13 17971232 10.1186/1750-2187-2-13 PMC2211295

[bib3] Auersperg N, Wong A, Choi K, Kang S, Leung P (2001) Ovarian surface epithelium: biology, endocrinology, and pathology. Endocrine rev 22: 255–288 11294827 10.1210/edrv.22.2.0422

[bib4] Cheng KW, Lahad J, Kuo W, Lapuk A, Yamada K, Auersperg N, Liu J, Smith-McCune K, Lu K, Fishman D, Gray J, Mills G (2004a) The RAB25 small GTPase determines aggressiveness of ovarian and breast cancers. Nat Med 10: 11251–11256 10.1038/nm1125 15502842

[bib5] Cheng KW, Lahad JP, Gray JW, Mills GB (2005) Emerging role of RAB GTPases in cancer and human disease. Cancer Res 65(7): 2516–2519. 15805241 10.1158/0008-5472.CAN-05-0573

[bib6] Cheng KW, Lahad JP, Kuo W-l, Lapuk A, Yamada K, Auersperg N, Liu J, Smith-McCune K, Lu KH, Fishman D, Gray JW, Mills GB (2004b) The RAB25 small GTPase determines aggressiveness of ovarian and breast cancers. Nat Med 10(11): 1251–1256 15502842 10.1038/nm1125

[bib7] Davies BR, Steele IA, Edmondson RJ, Zwolinski SA, Saretzki G, von Zglinicki T, O'Hare MJ (2003) Immortalisation of human ovarian surface epithelium with telomerase and temperature-sensitive SV40 large T antigen. Exp Cell Res 288(2): 390–402 12915130 10.1016/s0014-4827(03)00218-0

[bib8] Edmondson R, Monaghan J, Davies B (2002) The human ovarian surface epithelium is an androgen responsive tissue. Br J Cancer 86: 879–885 11953818 10.1038/sj.bjc.6600154 PMC2364138

[bib9] Enomoto T, Weghorst C, Inoue M, Tanizawa O, Rice J (1991) K-ras activation occurs frequently in mucinous adenocarcinomas and rarely in other common epithelial tumors of the human ovary. Am J Pathol 139(4): 777–785 1656759 PMC1886299

[bib10] Evangelou A, Jindal SK, Brown TJ, Letarte M (2000) Down-regulation of transforming growth factor {beta} receptors by androgen in ovarian cancer cells. Cancer Res 60(4): 929–935 10706107

[bib11] Evangelou A, Letarte M, Jurisica I, Sultan M, Murphy KJ, Rosen B, Brown TJ (2003) Loss of coordinated androgen regulation in nonmalignant ovarian epithelial cells with brca1/2 mutations and ovarian cancer cells. Cancer Res 63(10): 2416–2424 12750261

[bib12] Gaducci A, Gargini A, Palla E, Fanucchi A, Genazzani AR (2005) Polycystic ovary syndrome and gynecological cancers: is there a link? Gynecol Endocrinol 20(4): 200–208 16019362 10.1080/09513590400021201

[bib13] Gnanapragasam V, Robson C, Leung H, Neal D (2000) Androgen receptor signalling in the prostate. BJU Int 86: 1001–1013 11119093 10.1046/j.1464-410x.2000.00943.x

[bib14] Ilekis J, Connor J, Prins G, Ferrer K, Niederberger C, Scoccia B (1997) Expression of epidermal growth factor and androgen receptors in ovarian cancer. Gynecol Oncol 66(2): 250–254 9264571 10.1006/gyno.1997.4764

[bib15] Kouranti I, Sachse M, Arouche N, Goud B, Echard A (2006) Rab35 regulates an endocytic recycling pathway essential for the terminal steps of cytokinesis. Curr Biol 16(17): 1719–1725 16950109 10.1016/j.cub.2006.07.020

[bib16] Kuhnel R, de Graaff J, Rao B, Stolk J (1987) Androgen receptor predominance in human ovarian carcinoma. J Steroid Biochem 26: 393–397 3495702 10.1016/0022-4731(87)90106-3

[bib17] Leake R, Barnes D, Pinder S, Ellis I, Anderson L, Anderson T, Adamson R, Rhodes T, Miller K, Walker R (2000) Immunohistochemical detection of steroid receptors in breast cancer: a working protocol. J Clin Pathol 53(8): 634–635. 11002770 10.1136/jcp.53.8.634 PMC1762930

[bib18] Lee P, Rosen D, Zhu C, Silva E, Liu J (2005) Expression of progesterone receptor is a favorable prognostic marker in ovarian cancer. Gynecol Oncol 96(3): 671–677 15721410 10.1016/j.ygyno.2004.11.010

[bib19] Louro R, Nakaya H, Amaral P, Festa F, Sogayar M, da Silva A, Verjovski-Almeida S, Reis E (2007) Androgen responsive intronic non-coding RNAs. BMC Biol 5: 4 17263875 10.1186/1741-7007-5-4 PMC1800835

[bib20] Lukanova A, Kaaks R (2005) Endogenous hormones and ovarian cancer: epidemiology and current hypotheses. Cancer Epidemiol Biomarkers Prev 14(1): 98–107 15668482

[bib21] Lynch MA, Nakashima R, Song H, DeGroff VL, Wang D, Enomoto T, Weghorst CM (1998) Mutational analysis of the transforming growth factor {beta} receptor type. Cancer Res 58(19): 4227–4232 9766642

[bib22] Fujita M, Enomoto T, Yoshino K, Nomura T, Buzard GS, Inoue M, Okudaira Y (1995) Microsatellite instability and alterations in the *HMSH2* gene in human ovarian cancer. Int J Cancer 64(6): 361–366 8550235 10.1002/ijc.2910640602

[bib23] Motamed-Khorasani A, Jurisica I, Letarte M, Shaw P, Parkes R, Zhang X, Evangelou A, Rosen B, Brown T (2007) Differentially androgen-modulated genes in ovarian epithelial cells from BRCA mutation carriers and control patients predict ovarian cancer survival and disease progression. Oncogene 26: 198–214 16832351 10.1038/sj.onc.1209773

[bib24] Oldham W, Hamm H (2006) Structural basis of function in heterotrimeric G-proteins. Q Rev Biophys 39: 117 16923326 10.1017/S0033583506004306

[bib25] Risch H (1998) Hormonal etiology of epithelial ovarian cancer, with a hypothesis concerning the role of androgens and progesterone. J Natl Cancer Inst 90(23): 1774–1786 9839517 10.1093/jnci/90.23.1774

[bib26] Schildkraut JM, Schwingl PJ, Bastos E, Evanoff A, Hughes C (1996) Epithelial ovarian cancer risk among women with polycystic ovary syndrome. Obstetrics Gynecol 88(4 I): 554–559 10.1016/0029-7844(96)00226-8 8841217

[bib27] Wang PH, Chang C (2004) Androgens and ovarian cancers. Eur J Gynecol Oncol 25: 157–163 15032272

[bib28] Wilkinson SJ, Kucukmetin A, Cross P, Darby S, Gnanapragasam VJ, Calvert AH, Robson CN, Edmondson RJ (2008) Expression of gonadotrophin releasing hormone receptor I is a favorable prognostic factor in epithelial ovarian cancer. Hum Pathol 39(8): 1197–1204 18495208 10.1016/j.humpath.2007.12.011

[bib29] Yang F, Xiao-Yan X, Bi-Liang C, Xiang Dong M (2006) Knockdown of Rab25 expression by RNAi inhibits growth of human epithelial ovarian cancer cells *in vitro* and *in vivo*. Pathology 38(6): 561–567 17393986 10.1080/00313020601024037

